# Visible transparency modulated cooling windows using pseudorandom dielectric multilayers

**DOI:** 10.1515/nanoph-2024-0619

**Published:** 2025-02-07

**Authors:** Seok-Beom Seo, Jong-Goog Lee, Jae-Seon Yu, Jae-Hyun Kim, Serang Jung, Gumin Kang, Hyungduk Ko, Run Hu, Eungkyu Lee, Sun-Kyung Kim

**Affiliations:** Department of Applied Physics, Kyung Hee University, Yongin 17104, Republic of Korea; Department of Electronic Engineering, Kyung Hee University, Yongin 17104, Republic of Korea; Nanophotonics Research Center, Korea Institute of Science and Technology, Seoul 02792, Republic of Korea; KHU-KIST Department of Converging Science and Technology, Kyung Hee University, Seoul 02447, Republic of Korea; School of Energy and Power Engineering, Huazhong University of Science and Technology, Wuhan 430074, China

**Keywords:** passive cooling, multilayer, optical coating, machine learning, multispectral design, energy-saving window

## Abstract

The increasing global temperatures have escalated the demand for indoor cooling, thus requiring energy-saving solutions. Traditional approaches often integrate metal layers in cooling windows to block near-infrared (NIR) sunlight, which, albeit effective, lack the broad modulation of visible transmission and lead to heat accumulation due to sunlight absorption. Here, we address these limitations by developing cooling windows using ZnS/MgF_2_ multilayers, optimized through a binary optimization-based active learning process. We demonstrated that these multilayers, with a total thickness below 1 µm, effectively reduced indoor temperatures by blocking NIR sunlight while achieving desired visible transmittance. The designed multilayers exhibited visible transmittance ranging from 0.41 to 0.89 while retaining decent NIR reflectance between 0.37 and 0.52. These spectral characteristics remained consistent up to incident angles of >60°, ensuring their practical applicability for vertically oriented windows. Outdoor experiments showed substantial temperature reductions of up to 8.8 °C on floors compared to uncoated glass windows. The active learning-based multilayers exhibited superior performance compared to analytical ZnS/MgF_2_ distributed Bragg reflectors with equivalent thicknesses by improving NIR reflectance and modulating visible transmittance. In addition, multilayers with a greater number of bits extensively tuned transmission color, enabling customization for aesthetic purposes. These findings suggest that all-dielectric multilayers can provide a scalable, cost-effective alternative for reducing energy consumption in buildings and vehicles with large glass surfaces, supporting efforts to mitigate climate change through enhanced energy efficiency.

## 1 Introduction

In recent years, the global rise in temperature during summer, largely attributed to climate change, has significantly increased the demand for indoor cooling [1]. This surge in energy consumption exacerbates global warming, repeating a detrimental cycle. Mitigating this increased energy demand is partially achievable through the use of energy-saving (i.e., cooling) windows [2], [3], [4], [5], [6], [7], [8], [9], [10], [11]; such windows are particularly beneficial in buildings and automobiles with large window-to-wall area ratios, where they can substantially save cooling energy.

Radiative cooling operates by emitting thermal radiation from hot objects into outer space through the atmospheric window (8–14 μm in wavelength) [12], [13], [14]. This passive cooling approach is based on two critical principles. First, the emission of thermal radiation within the atmospheric window prevents heat energy from returning to the ground. Recent studies on cooling vertical windows and sidewalls have considered directional thermal emission. These are categorized based on the symmetry of thermal radiation distribution: symmetric [15], [16], [17] and asymmetric [18], [19], [20]. Thermal emitters with symmetric side thermal radiation can provide thermal comfort to users exposed to personal electronic device [16]. Also, they help mitigate the greenhouse effect between dense urban buildings [15]. In comparison, asymmetric thermal emitters that block ground-facing thermal radiation enhance the cooling performance compared to omnidirectional thermal emitters, when applied to vertical surfaces in hot climates [18], [19], [20]. Secondly, sunlight (0.3–2.5 μm in wavelength) must be blocked to avoid heat generation. Glass windows, whether in buildings or automobiles, strongly emit thermal radiation within the atmospheric window due to the inherent phonon resonances of silica [21]. However, they remain transparent within the solar spectrum. As a result, cooling windows are primarily designed to block near-infrared (NIR) light, which constitutes approximately 0.48 of solar energy, serving as a major source of indoor heating [4]. Therefore, we aim to improve cooling efficiency within buildings by blocking the NIR component of sunlight in a vertical configuration, rather than relying solely on radiative cooling performance. Previous studies have explored the integration of ultrathin (approximately 10 nm) silver layers into glass windows to block NIR light within the entire range of 0.78–2.5 μm [5], [6], [7]. While these attempts achieve nearly complete NIR blocking, they are accompanied by a reduction in visible transmission. Furthermore, the ultrathin silver layers absorb both visible and NIR light to some extent, leading to indoor heat accumulation.

To overcome these limitations, Zhu et al. developed an all-dielectric multilayer for cooling windows, using SiO_2_ and TiO_2_ [4]. Their design incorporated two distinct distributed Bragg reflectors (DBR) for NIR reflection in the 0.78–1.4 μm range. Despite employing only two dielectric materials, this approach required stacking 30 layers, resulting in a total thickness exceeding 3 μm. In contrast, Yu et al. reported a cooling window with high visible transmittance (0.86) and NIR reflectance (0.48) using ZnS and MgF_2_, with a total thickness of 680 nm [2]. By stacking five layers of ZnS/MgF_2_ on a glass window, their design exhibited substantial cooling performance; outdoor experiments using black absorbers through the cooling window showed a temperature reduction of up to 12.7 °C compared to an uncoated window.

**Figure 1:**
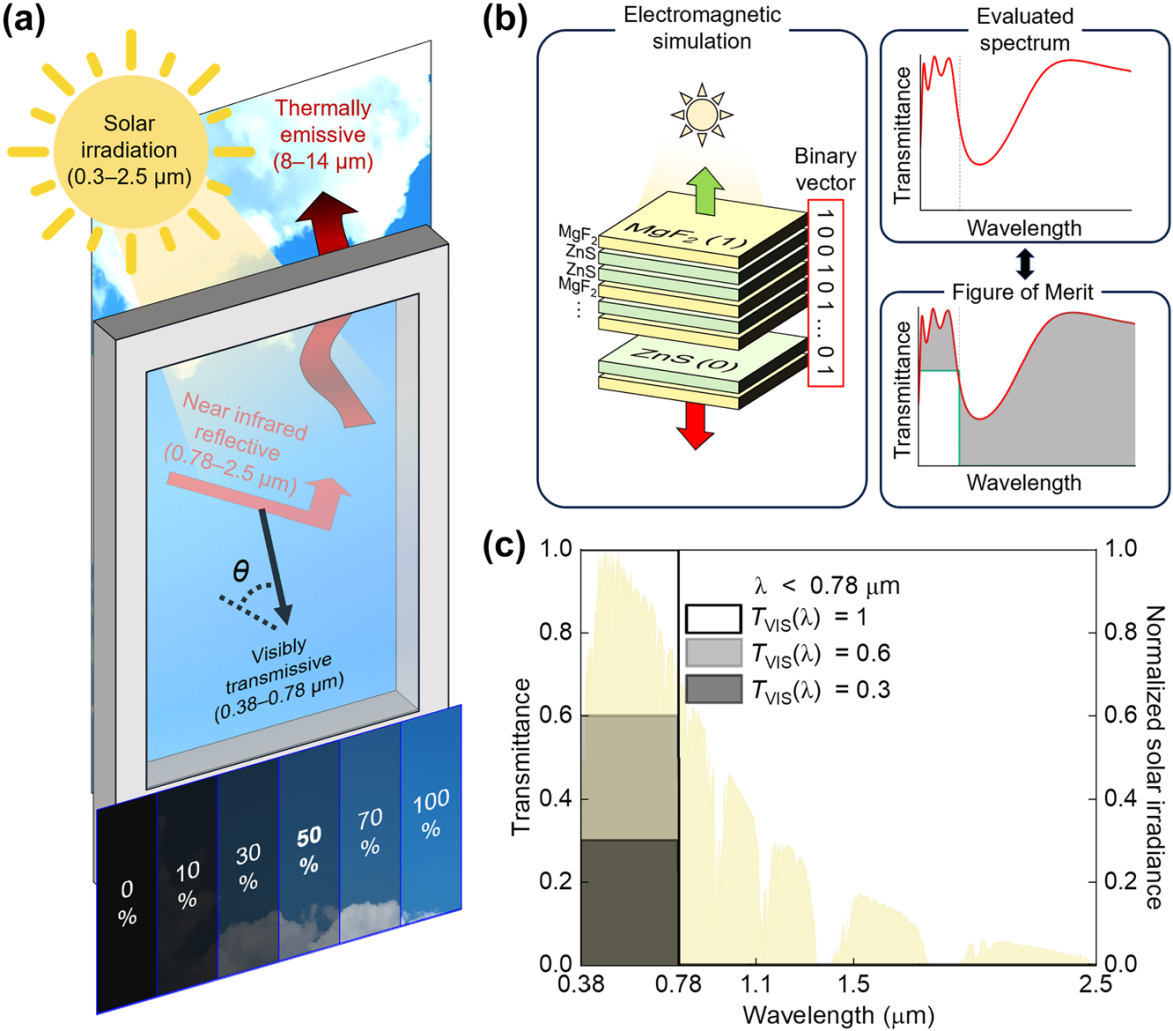
Concept and performance of cooling windows. (a) Conceptual schematic of visible transmittance modulated cooling windows. (b) Schematic of the used active learning-based multilayer design. (c) Target spectra (*T*_VIS_(*λ*) = 1, 0.6, and 0.3) to evaluate the FoM, each with zero NIR (0.78–2.5 µm) transmission and modulated visible (0.38–0.78 µm) transmittance of 1, 0.6, and 0.3. The yellow background indicates normalized AM1.5G solar irradiance.

Inspired by the previous approach [2], we designed a cooling window with adjustable visible transmittance (Figure 1(a)). Using the identical material set of ZnS and MgF_2_, we developed cooling windows with visible transmittance ranging from 0.41 to 0.89, while maintaining NIR reflectance between 0.37 and 0.52. Outdoor daytime experiments revealed that one of our designs, with 0.41 visible transmission and 0.37 NIR reflection, reduced floor temperatures by 8.8 °C, compared to an uncoated window. Notably, our cooling window coatings have total thicknesses of less than 700 nm and consist of fewer than eight layers, which minimizes the potential for thermal mismatch. However, delamination or cracking issues related to thermal mismatch may arise when the total thickness of a multilayer exceeds one micron [22]. In such cases, the insertion of an ultrathin (∼10 nm) adhesive layer, such as Y_2_O_3_, at each interface is necessary.

## 2 Results and discussion

### 2.1 Binary optimization-based active learning

To design ZnS/MgF_2_ multilayers for cooling windows, we employed a binary optimization-based active learning model [22], [23], [24], [25], [26]. The material dispersions of the used ZnS and MgF_2_ are shown in Figure S1. In this model, the two dielectric layers forming multilayers were encoded into binary digits (e.g., 0 for ZnS and 1 for MgF_2_), as illustrated in Figure 1(b). Consequently, a given multilayer was represented by a specific binary vector. The transfer matrix method (TMM) was used to decode these binary vectors into multilayers and to determine their reflection and transmission spectra within the 0.38–2.5 µm range. The exact electromagnetic simulation results were quantified as a metric to evaluate the fulfillment of the optimization objectives, termed the figure of merit (FoM). To design optimal cooling windows, we defined the FoM using the following equation:

$$\text{FoM}=\frac{\int_{0.38\;\mu \text{m}}^{2.5\;\mu \text{m}}{\left[I_{\text{sol}}\left(\lambda \right)\left\{T_{\text{tar}}\left(\lambda \right)-T\left(\lambda \right)\right\}\right]}^{2}\text{d}\lambda }{\int_{0.38\;\mu \text{m}}^{2.5\;\mu \text{m}}{\left|I_{\text{sol}}\left(\lambda \right)\right|}^{2}\text{d}\lambda }$$

where *T*_tar_(*λ*) represents the target transmission spectrum and *I*_sol_(*λ*) denotes the AM1.5G solar irradiance, as shown in Figure 1(c). *T*(*λ*) is the evaluated transmission spectrum obtained through TMM. In this equation, the evaluated spectrum was subtracted from the target spectrum and a weighting factor was applied based on the AM1.5G solar irradiance. This approach ensures that the evaluated spectrum aligns with the target spectrum, with a greater emphasis on wavelengths with high solar irradiance (0.4 µm ≤ *λ* ≤ 1.1 µm).

For a binary vector with *N* digits, the parametric space contains 2^*N*^ elements, including the calculation of FoMs for all possible binary vectors. Rather than exhaustively computing each case, we generated 25 random binary vectors and evaluated their FoMs to prepare an initial training dataset [22], [23]. Using this dataset, factorization machines (FM) were employed to construct a surrogate model. Instead of evaluating 2^*N*^ iterations, active learning examines a limited number of points (typically, 1,000) and infers a function by linking these data points to approximate the actual parametric space. This inferred function is referred to as the surrogate model. Subsequently, quadratic unconstrained binary optimization (QUBO) was solved by brute force to predict a new binary vector that yields a minimum FoM. Brute force evaluates every possible solution within the parameter space, ensuring the identification of the global optimum within a given machine learning model [27]. However, it is computationally expensive and impractical for large-scale problems with extensive parameter spaces. Then, the TMM calculated the FoM of this new binary vector, which was added to the training dataset. This iterative approach achieves higher precision; the predicted binary vectors converge towards a quasi-optimal point in the parametric space, thereby decreasing their FoMs to the lowest possible values.

The one-bit thickness (*t*_bit_) and the total number of bits (*N*) must be appropriately determined. Setting *t*_bit_ to be less than or equal to one-tenth of the target wavelength (*t*_bit_ ≤ *λ*/10) ensures precise phase control, thereby meeting the complicated interference conditions. In addition, both computational resources and economic viability must be considered when defining *N*. In this study, we set *N* to 16–18 and *t*_bit_ to 35 nm, resulting in a total thickness of *t*_bit_ × *N* = 560–630 nm.

### 2.2 Modulation of visible transmission

**Figure 2:**
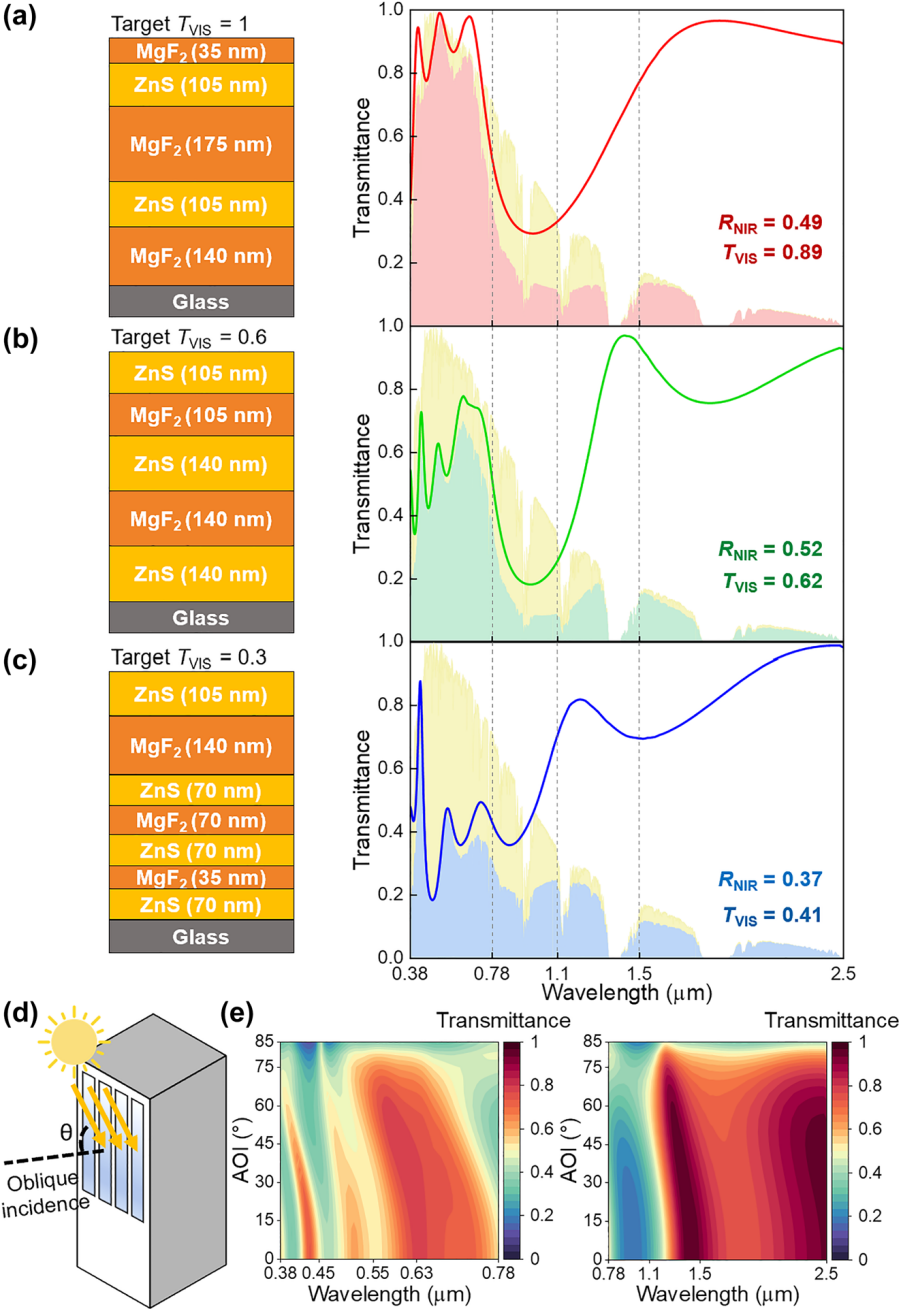
Designed cooling windows with adjustable visible transmittance. (a–c) ZnS/MgF_2_ multilayer configurations (left) and their transmittance spectra across the solar spectrum (upper), for the cases with (a) *T*_VIS_ = 1, (b) *T*_VIS_ = 0.6, and (c) *T*_VIS_ = 0.3. The colored (red, green, and blue) shaded areas represent normalized, AM1.5G solar irradiance weighted transmittance. (d) Illustration of a vertically oriented window. (e) Visible and NIR transmittance spectra for the multilayer-coated window with target *T*_VIS_ = 0.6 at varying AOI from 0° to 85°.

We designed three ZnS/MgF_2_ multilayers with distinct visible transmittance, identified through the active learning scheme. Their detailed configurations and transmission spectra are illustrated in Figure 2(a)–(c). Each multilayer was designed to achieve target visible transmittance of 1, 0.6, or 0.3, denoted as *T*_VIS_ = 1, *T*_VIS_ = 0.6, and *T*_VIS_ = 0.3, respectively. Notably, the configurations for *T*_VIS_ = 1 and *T*_VIS_ = 0.3 were designed with a 16-bit condition (*N* = 16), whereas *T*_VIS_ = 0.6 designed with an 18-bit (*N* = 18) condition. In each graph, the red, green, and blue solid lines represent the transmission spectra of the cooling windows across the visible-to-NIR spectrum, while the yellow background indicates the AM1.5G solar irradiance. The colored shaded areas correspond to the portions of the AM1.5G solar irradiance that pass through the cooling windows. The average transmittance and reflectance values for the visible (0.38–0.78 µm) and NIR (0.78–2.5 µm) spectra are displayed in the lower right corner of each graph.

The designed multilayers were constructed by stacking ZnS/MgF_2_ layers in a pseudorandom (i.e., non-intuitive) manner. While these multilayers maintained decent NIR reflectance (*R*_NIR_) between 0.37 and 0.52, with a local minimum in the 0.78–1.1 µm, their visible transmittance values were sequentially modulated from 0.89 to 0.41. The NIR reflection is primarily determined by the index contrast of the constituent materials in a multilayer and its total thickness, which are nearly identical for the three multilayers. Notably, for visible transmission, the target and designed values exhibited slight differences for the *T*_VIS_ = 1 and *T*_VIS_ = 0.3 cases, which could be addressed by employing using diverse (i.e., quaternary) materials and/or a great number of bits [18].

Although the three multilayers were initially designed considering only normal incidence, in practical scenarios, sunlight typically strikes outdoor objects at oblique angles (Figure 2(d)) [2], [25], [28]. Due to their consistent transmittance and reflectance across a range of visible and NIR wavelengths, these multilayers demonstrated robustness to variations in the angle of incidence (AOI) (Figures 2(e) and S2). For all the multilayer configurations, the transmission and reflection spectra remained nearly constant up to AOIs of 60° and 80°, respectively, indicating their practical use for vertical windows in building and automobiles.

### 2.3 Fabrication and optical characterization

**Figure 3:**
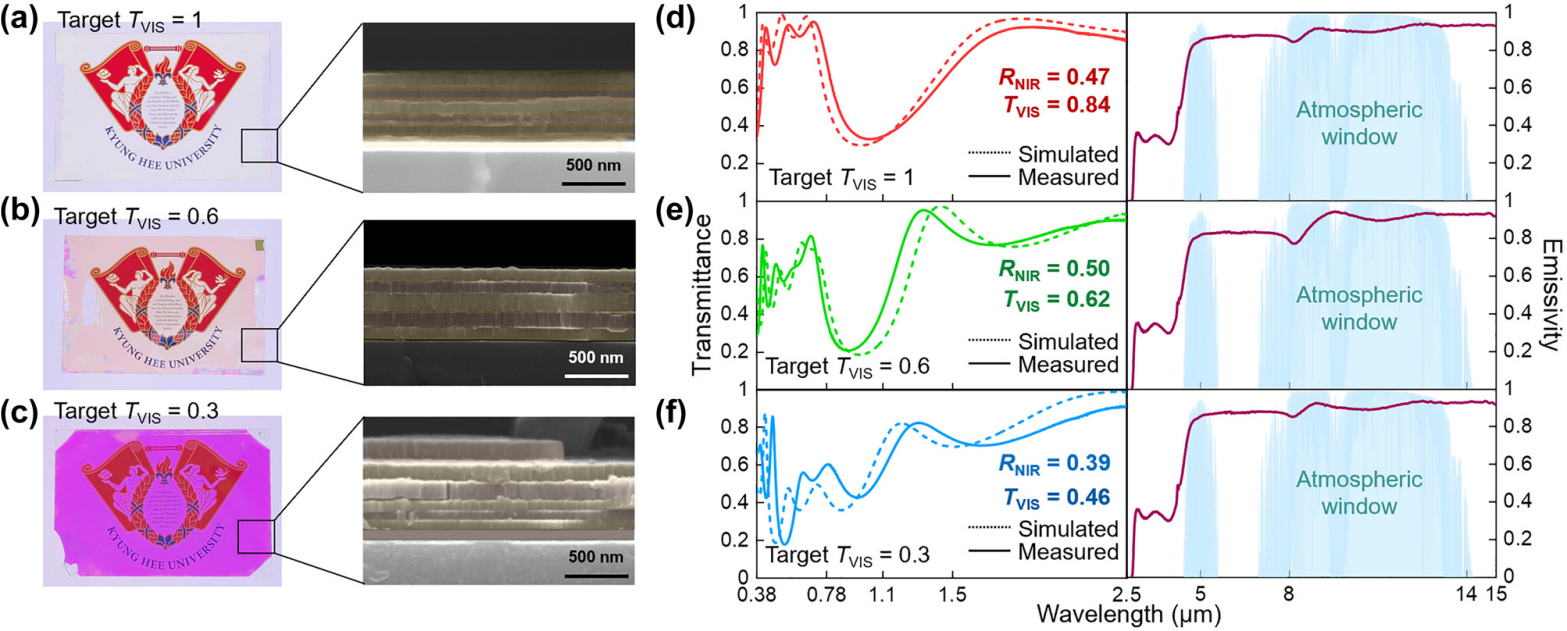
Optical characterization of fabricated cooling windows. (a–c) Photographs (left) and cross-sectional SEM images (right) of fabricated cooling windows, with (a) *T*_VIS_ = 1, (b) *T*_VIS_ = 0.6, and (c) *T*_VIS_ = 0.3. (d–f) Left: measured (solid lines) and simulated (dashed lines) transmission spectra of the same samples with (d) *T*_VIS_ = 1, (e) *T*_VIS_ = 0.6, and (f) *T*_VIS_ = 0.3 within the solar spectrum. Right: measured emissivity of the same samples. The blue background areas represent the atmospheric window.

We fabricated the designed ZnS/MgF_2_ multilayers on glass windows using a thermal evaporator. Commercial soda-lime glass with dimensions of 76 mm × 52 mm and with a thickness of 1 mm was used as the substrate. The multilayer coating covered nearly the entire surface of the glass, except for a small, masked area. Figure 3(a)–(c) presents both the camera and cross-sectional scanning electron microscopy (SEM) images of the fabricated samples with *T*_VIS_ = 1, *T*_VIS_ = 0.6, and *T*_VIS_ = 0.3. Figure 3(d)–(f) displays the measured and simulated transmission spectra of the fabricated samples in both the solar (left) and thermal radiation (right) spectra. Consistent with the results of the corresponding designs, the cooling windows exhibited visible transmittance values of 0.84, 0.62, and 0.47, and NIR reflectance values of 0.47, 0.50, and 0.39, respectively. The actual total thicknesses of the multilayers ranged from 560 to 630 nm, which is less than one-tenth of the wavelengths of thermal radiation. The three multilayers with a submicron thickness preserved the emissivity of bare glass (0.87), with measured emissivity values of 0.91, 0.90, and 0.91, respectively. These results suggest that the multilayer-coated windows maintain their cooling performance by effectively emitting thermal radiation.

### 2.4 Outdoor daytime experiments

**Figure 4:**
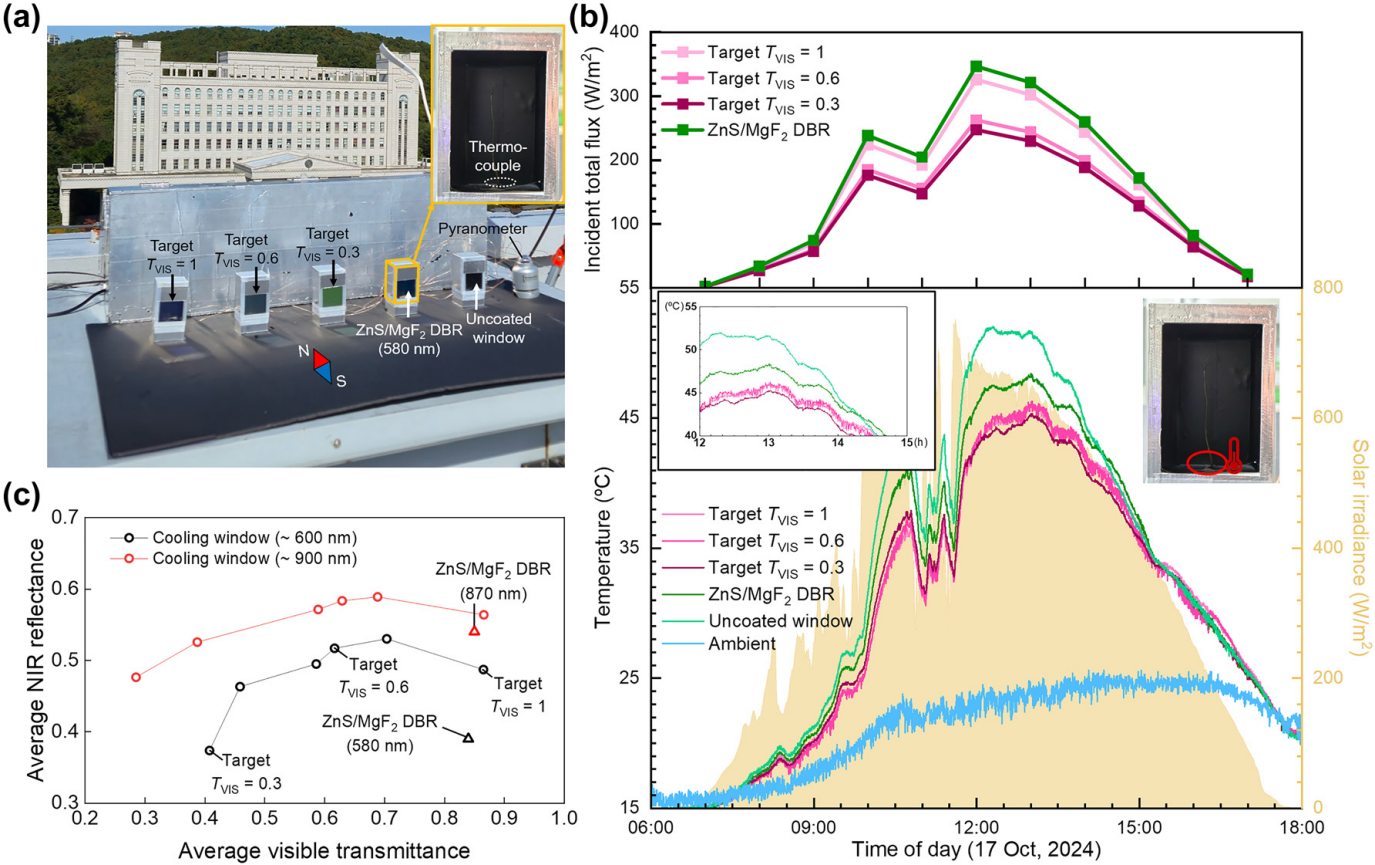
Outdoor cooling performance. (a) Photograph of the used measurement setup with miniaturized houses. (b) Top: estimated solar energy flux transmitted through each window based on its transmittance spectrum. Bottom: measured floor temperatures of each miniaturized house and ambient temperatures. Inset: measured temperatures between 12:00 pm and 3:00 pm. (c) Visible transmittance and NIR reflectance of various cooling windows including those used in the outdoor experiments and ZnS/MgF_2_ DBRs with the corresponding thicknesses.

To assess the cooling performance of the multilayer-coated glass windows, an outdoor experiment setup was established on a building rooftop (Figure 4(a)). The outdoor measurement setup illustrated in Figure 4(a) is described in more detail in Figure S4. For comparison, we prepared an uncoated glass window and a glass window coated with ZnS/MgF_2_ DBR. The DBR thickness was approximately 600 nm to ensure a fair comparison, matching closely with the thicknesses of the multilayers (Figure S3). The DBR was designed to maximize the average NIR reflectance by centering the wavelength at 950 nm. The exterior and interior surfaces of the miniaturized houses were covered with aluminium tape and black insulating tape, respectively, with black foam boards positioned underneath the miniaturized houses. Thermocouples were employed to measure the floor temperatures by being placed on the floor (Figure 4(b)). We obtained the floor temperature rather than the indoor air temperature. In buildings, the primary sources of heat influencing indoor air temperature are the heated floor and sidewalls, which implies that indoor temperatures follow the same trend as floor temperatures. However, the variability in indoor volume, window coverage ratio, absorptivity of the floor and sidewalls, and the level of convection across different buildings complicates the relationship between floor and indoor air temperatures. The vertical windows were oriented toward the south. Considering the time-varying AOI of direct sunlight, estimated visible transmittance and NIR reflectance were calculated for all the samples (Figure S5).

The multilayer-coated windows demonstrated superior cooling performance compared to the uncoated and DBR-coated windows. At 12:30 pm, the sample with *T*_VIS_ = 0.6 achieved maximum temperature reduction of 8.8 °C and 5.4 °C compared to the uncoated and DBR samples, respectively for the floor settings. Notably, the differences in floor temperature among the multilayer-coated windows were within merely 1 °C, which is attributed to the trade-off between visible light modulation and NIR blocking performance (Figures 3(d)–(f) and S5). For instance, the sample with *T*_VIS_ = 0.3 exhibited less NIR reflection than the other two samples. Although the floor and indoor temperatures are expected to be closely related, quantifying their relationship was challenging due to numerous complex factors, such as building scale, ventilation levels, and conduction or convection coefficients [14], [21], [24]. Consequently, measuring the indoor temperature within our miniaturized houses may not yield practical results, as the setup does not accurately replicate a real building environment.

Figure 4(c) summarizes the visible transmittance and NIR reflectance, two key performance metrics, for various multilayers designed through the active learning scheme, including those used in the outdoor experiments. We categorized them into two groups based on their total thicknesses, constrained to approximately 600 and 900 nm. For comparison, ZnS/MgF_2_ DBRs with a center wavelength of 950 nm were also plotted. Both multilayer groups, differing in thickness, encompassed a broad range of visible transmittance values between approximately 0.3 and 0.9. Notably, the thicker multilayers, designed with a greater number of bits, exhibited higher NIR reflectance, exceeding that of DBRs with an equivalent thickness. These findings highlight the advantages of active learning-based optical design over analytical optical formulas in addressing multispectral problems.

### 2.5 Tuning of transmission color

**Figure 5:**
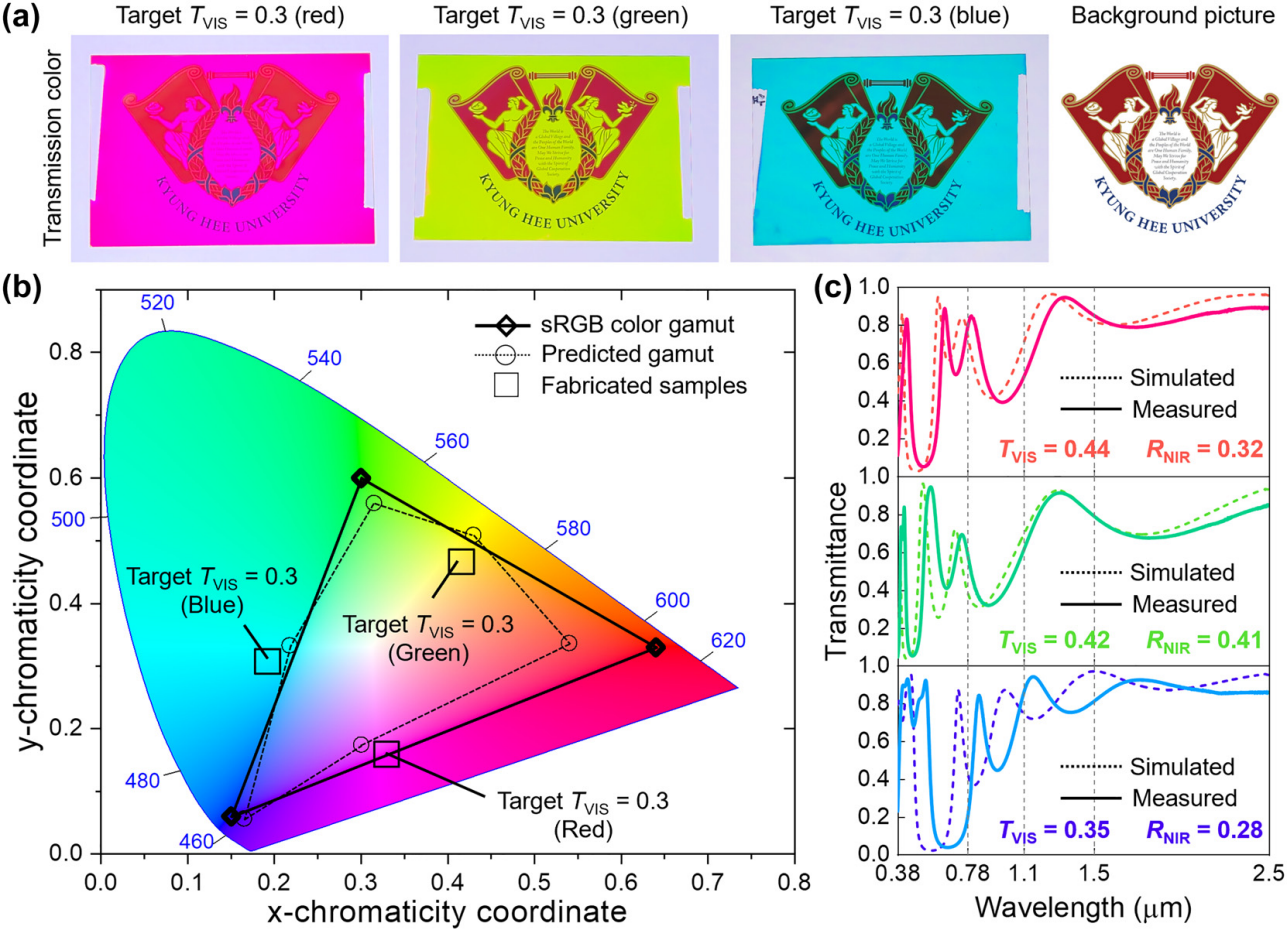
Colored cooling windows. (a) Colored cooling windows with *T*_VIS_ = 0.3, showing three different transmission color (red, green, and blue). (b) Predicted color gamut (empty circles and dashed contour) defined by six colors (red, green, blue, cyan, magenta, and yellow) of designed colored cooling windows with *T*_VIS_ = 0.3. Diamond symbols and triangular solid contour represents the sRGB color gamut. (c) Measured transmission spectra of the colored cooling windows depicted in the Figure 5(a).

With enhanced computational resources, a greater number of bits can be allocated to improve NIR reflection and to achieve higher precision in tuning visible transmission. Moreover, customization of the desired color, for both transmission and reflection, can be accomplished by incorporating a color-associated factor into the FoM equation [3], [29], [30], [31]. Figure 5(a) displays red, green, and blue cooling windows with *T*_VIS_ = 0.3. Under a condition of *N* = 20, these cooling windows achieved transmission colors within the specific regions in the CIE 1931 color space, as shown in Figure 5(b). The boundary of the color gamut was determined from the colors of six optimally colored cooling windows, each targeting red, green, blue, cyan, magenta, and yellow. By employing a larger *N* or weighting color precision within the FoM equation, cooling windows can be produced with more vivid and precise colors. Figure 5(c) presents the transmission spectra of each colored cooling window, revealing intricate spectral characteristics compared to previous cooling glass designs (Figure 3(d)–(f)). Notably, these results were achieved using only transparent dielectrics, without the use of absorptive metals. The objective (i.e., FoM) for the fabricated samples shown in Figure 3 was initially focused solely on achieving a specific level of visible transmittance (*T*_VIS_) while maximizing NIR reflectance. The selection of the cooling window’s color was incidental. However, the active learning process allows for the identification of multiple solutions that meet the objective within an acceptable tolerance range. We incorporated a color coordinate into the FoM to facilitate the selection of the desired color (Figure 5). Based on simulation results, we demonstrated achromatic cooling windows that provide balanced suppression of transparency across the visible spectrum (Figure S6).

The key advantage of our design is its ability to nearly sustain the cooling performance achieved by blocking the NIR component of solar irradiance, even with variations in transmitted color. There is, however, a slight trade-off associated with different colors (Figure 5(c)), particularly with blue, which requires the suppression of transmittance for green and red light (0.5–0.78 µm). For blue colored cooling window, NIR light must be blocked within the range of 0.78–1.1 µm, which necessitates an extension of the high reflectance wavelength range from 0.5 to 1.1 µm. However, suppressing the green-to-red spectral region inevitably leads to an increase in closely spaced NIR transmittance. This phenomenon arises fundamentally because our cooling window coatings are reminiscent of distributed Bragg reflectors (DBR), where their reflection bandwidth is dictated by the refractive index contrast between the two stacked materials (i.e., ZnS and MgF_2_). In our cooling window coatings, a slight perturbation was applied to the DBR configuration, enabling the achievement of desired spectral characteristics within specific wavelength ranges. This minor modification forms the basis for our designation of the cooling window coatings as pseudorandom multilayers.

## 3 Conclusions

We successfully designed cooling windows with varying levels of visible transmission by strategically stacking a limited number of dielectric layers, ensuring that the total thickness remains below 1 µm. These cooling windows reduced indoor heating by blocking NIR sunlight. Although the temperature reduction achieved was within a few degrees, this approach is cost-effective and scalable, making it a practical solution for widespread applications. Notably, the designs were accomplished without the use of metal layers. For applications requiring enhanced cooling performance, the design process can be further refined by employing more bits in the binary optimization-based active learning scheme, which would lead to improved NIR reflection and greater reductions in indoor temperature. These advancements highlight the potential for optimizing all-dielectric cooling windows through computational design without resorting to metal incorporation.

## Supplementary Material

Supplementary Material Details
