# Supplementary material for: Visible transparency modulated cooling windows using pseudorandom dielectric multilayers
Source: Nanophotonics. 2025 Feb 7;14(10):1587–95. doi: 10.1515/nanoph-2024-0619 (PMC12116263; doi:10.1515/nanoph-2024-0619)
Supplement: Supplementary file 1 — Supplementary Material Details [file j_nanoph-2024-0619_suppl_001.docx]

***Supporting Information for***

**Visible transparency modulated cooling glass using pseudorandom dielectric multilayers**

Seok-Beom Seo^1+^, Jong-Goog Lee^1+^, Jae-Seon Yu^1^, Jae-Hyun Kim^1^, Serang Jung^2^, Gumin Kang^3^, Hyungduk Ko^3,4^, Run Hu^1,5^, Eungkyu Lee^2*^, Sun-Kyung Kim^1^*

^1^Department of Applied Physics, Kyung Hee University, Yongin 17104, Republic of Korea

^2^Department of Electronic Engineering, Kyung Hee University, Yongin 17104, Republic of Korea

^3^KHU-KIST Department of Converging Science and Technology, Kyung Hee University, Seoul 02447, Republic of Korea

^4^Nanophotonics Research Center, Korea Institute of Science and Technology, Seoul 02792, Republic of Korea

^5^School of Energy and Power Engineering, Huazhong University of Science and Technology, Wuhan 430074, China

^+^These authors contributed equally to this study.
^*^Corresponding authors: [eleest@khu.ac.kr](mailto:eleest@khu.ac.kr), sunkim@khu.ac.kr

**This PDF file includes the following sections:**

Methods

Supplement Figures S1-S6

**Methods**

**Design optimization:** In the optimization design process, a surrogate function was formulated using the Factorization Machine (FM) model within the “xLearn” package. The hyperparameters of the FM model were optimized by minimizing the loss function through stochastic gradient descent with a learning rate of 0.001 and an L2 regularization parameter of 0.001. The number of epochs was set to 20,000, with early stopping applied to prevent overfitting. Of the training dataset, 80% was used for supervised learning, and the remaining 20% was reserved for cross-validation. Active learning was conducted on a workstation equipped with an AMD Ryzen® Threadripper™ PRO 5945WX 16-core processor and 512GB of DDR4 3200MHz ECC DIMM RAM (8 x 64GB).

**Fabrication:** Multilayer and Distributed Bragg Reflector (DBR) coatings were fabricated on a glass substrate (0350-0001, LK Lab Korea), while thin films were deposited on a silicon substrate (thickness 500 ± 30 μm, Hi-Solar Co. Ltd.) for optical and material characterizations. Both glass and silicon substrates were sequentially cleaned with acetone, isopropyl alcohol, and distilled water in an ultrasonic bath. After cleaning, a soft bake at 150 °C for 10 minutes was applied to remove residual moisture and enhance adhesion. Thin films of ZnS (iTasco, 99.99%) and MgF₂ (iTasco, 99.99%) were deposited via thermal evaporation with a deposition rate of 2 Å/s for both materials. Substrate temperature was maintained at room temperature throughout deposition.

**Optical characterization:** The refractive indices of MgF₂ and ZnS thin films were measured across visible and near-infrared wavelengths using ellipsometry (IR-VASE, J. A. Woollam Co.). Film thicknesses were measured with a surface profilometer (Alpha-step D-500, KLA-Tencor). The transmittance spectrum of the cooling windows was obtained using a spectrophotometer (Cary 5000, Varian), while the absorption spectrum was recorded with an FTIR spectrometer (INVENIO R, Bruker) equipped with an HgCdTe (MCT) detector (covering the 1.6–14 μm range). Cross-sectional SEM images of the samples were acquired using an FE-SEM (S-4800, Hitachi) after Pt coating the samples.

**Outdoor measurements:** Outdoor measurements were conducted on the rooftop of the College of Electronics & Information building at Kyung Hee University, Suwon-si, Gyeonggi-do, South Korea (coordinates: 37.23983° N, 127.08371° E). Thermocouples (SA1-K-SC, Omega™) logged real-time sample temperatures via a (specify model) temperature logger. Solar irradiance and ambient temperature were measured using a pyranometer (SR05-D1A3, Hukseflux). An indoor simulator was constructed with a PLA body, black insulating tape, and aluminum tape for sunlight reflection; PLA parts were 3D-printed.


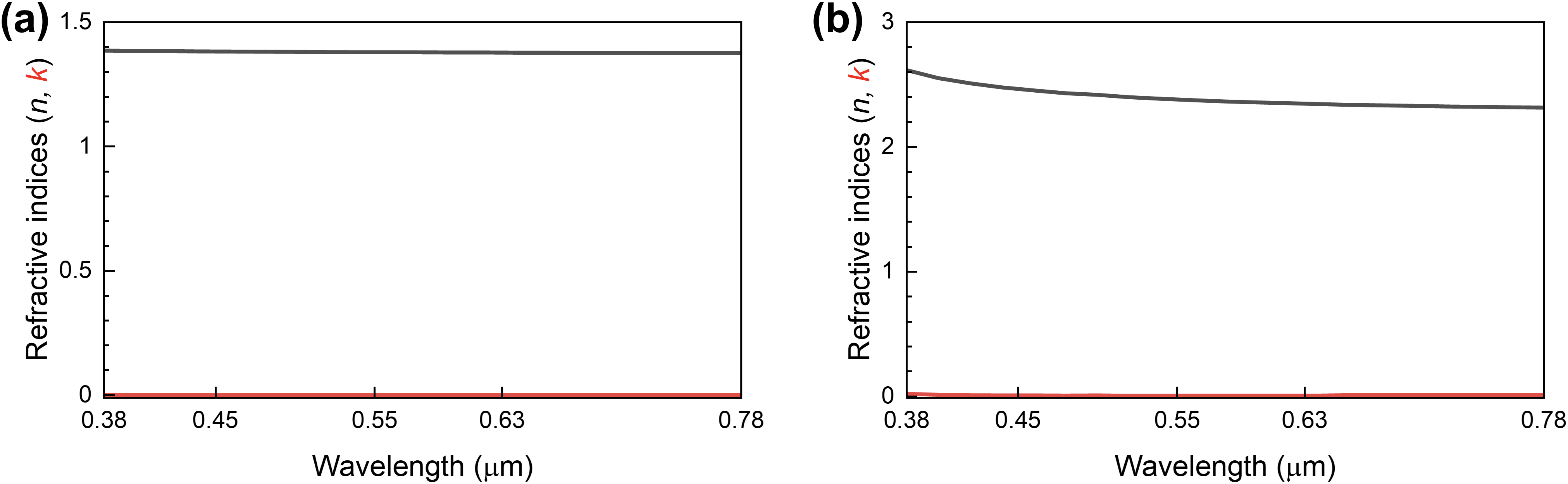


**Figure S1.** **Refractive indices of MgF_2_ and ZnS.** (a, b) Measured Refractive indicies of (a) MgF_2_ and (b) ZnS. A thermal evaporator was used for thin film deposition and a spectroscopic ellipsometer was used for refractive index measurements.


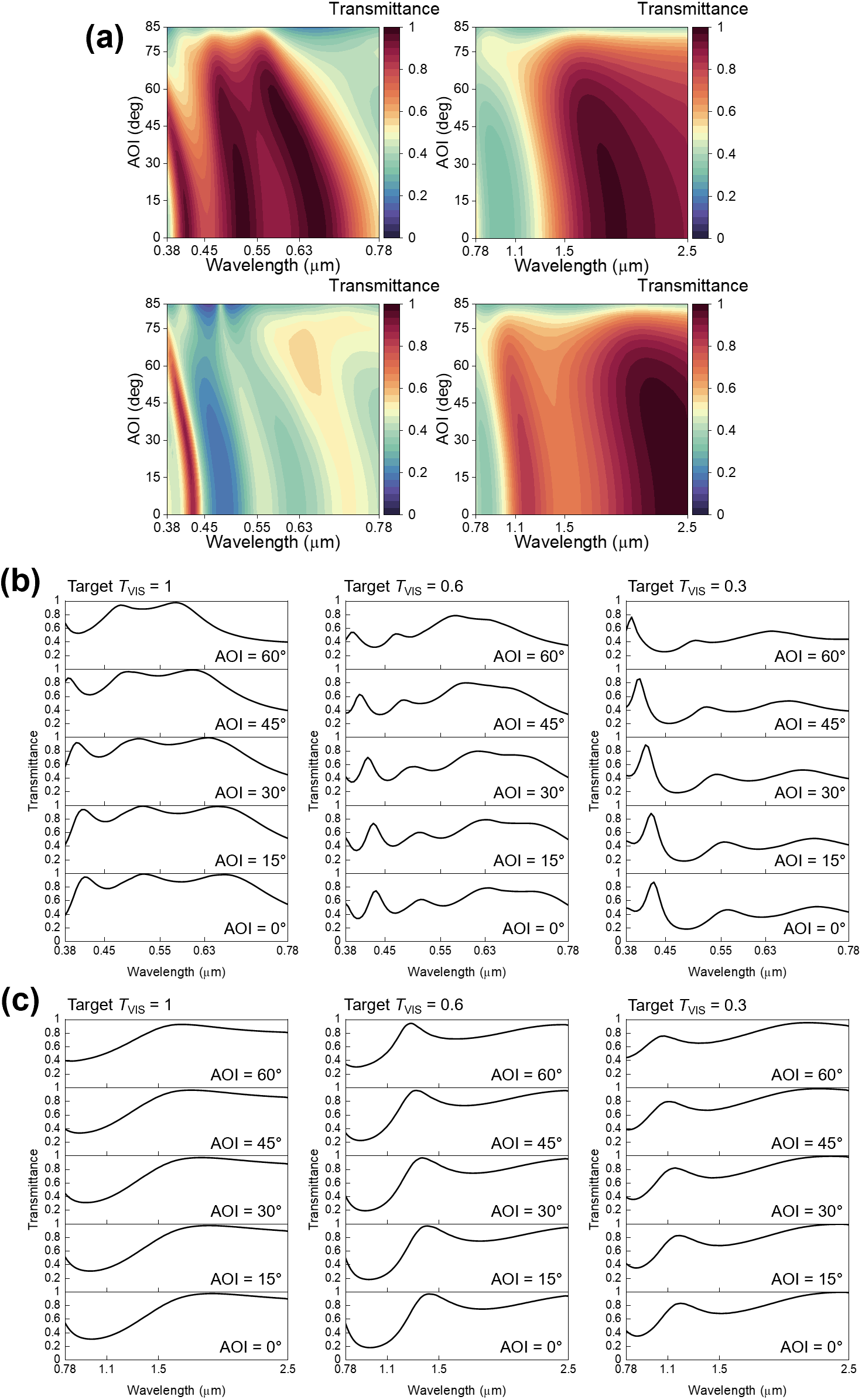


**Figure S2.** **AOI dependent transmittance spectra** (a) AOI dependent visible (left) and NIR (right) transmittance spectra of the multilayer-coated windows with *T*_VIS_ = 1 (Figure 2(a)) and target *T*_VIS_ = 0.3 (Figure 2(c)). (b-c) Transmittance spectra of the three samples (Figure 2(a-c)) at specific AOI values (0°, 15°, 30°, 45°, and 60°).


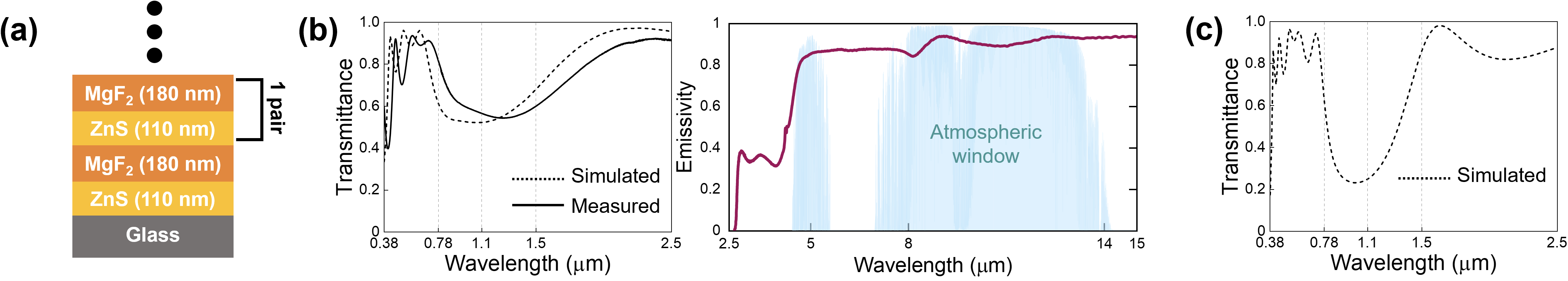

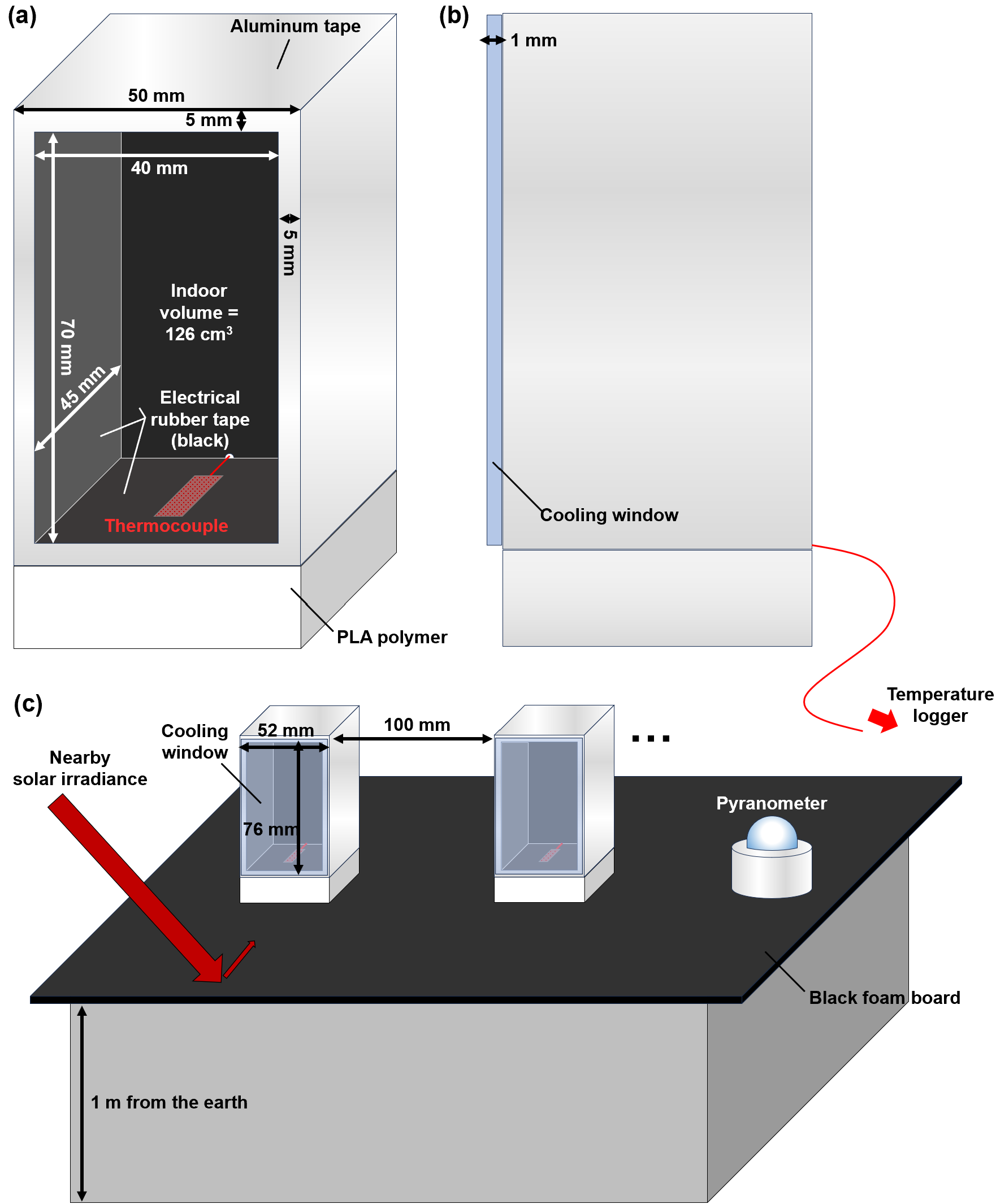

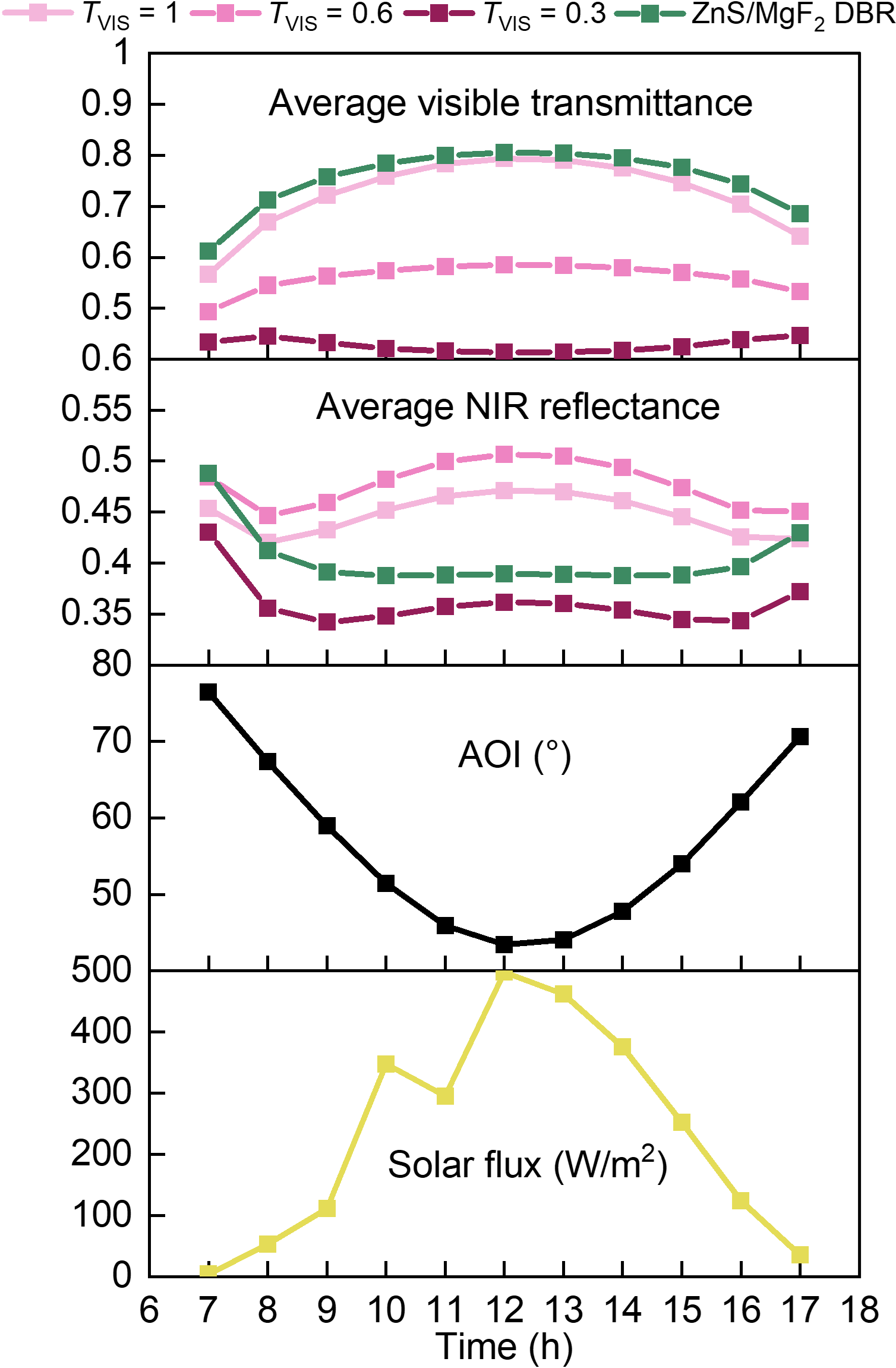


**Figure S3. Optical characterization of ZnS/MgF_2_ DBRs.** (a) Schematic of a ZnS/MgF_2_, designed for achieving a center wavelength of 950 nm. (b) Optical characteristics of a glass window coated with a 2-pair ZnS/MgF_2_ DBR having a total thickness of 580 nm. Left: Simulated (dashed line) and measured (solid line) transmittance spectra within the solar spectrum (0.38–2.5 µm). Right: Measured emissivity within the thermal radiation spectrum (2.5–15 µm). (c) Simulated transmittance spectrum of a 3-pair ZnS/MgF_2_ DBR with a thickness of 870 nm within the solar spectrum.

**Figure S4. Detailed measurement setup for outdoor daytime experiments.** (a) Oblique view of the indoor simulator equipped with the cooling window. (b) Side view of the indoor simulator equipped the cooling window. The indoor simulators were surrounded by black foam board to eliminate additional light reflected from the ground. The setup was elevated by 1 meter above the ground to block thermal emissions from the ground.

**Figure S5. Detailed information of the outdoor daytime experiments.** Time-varying visible transmittance and NIR reflectance of the used samples in Figure 4, together with the AOIs and solar energy flux of direct sunlight. The solar energy flux was calculated by multiplying cosine values of AOIs with the measured hourly solar energy flux values.


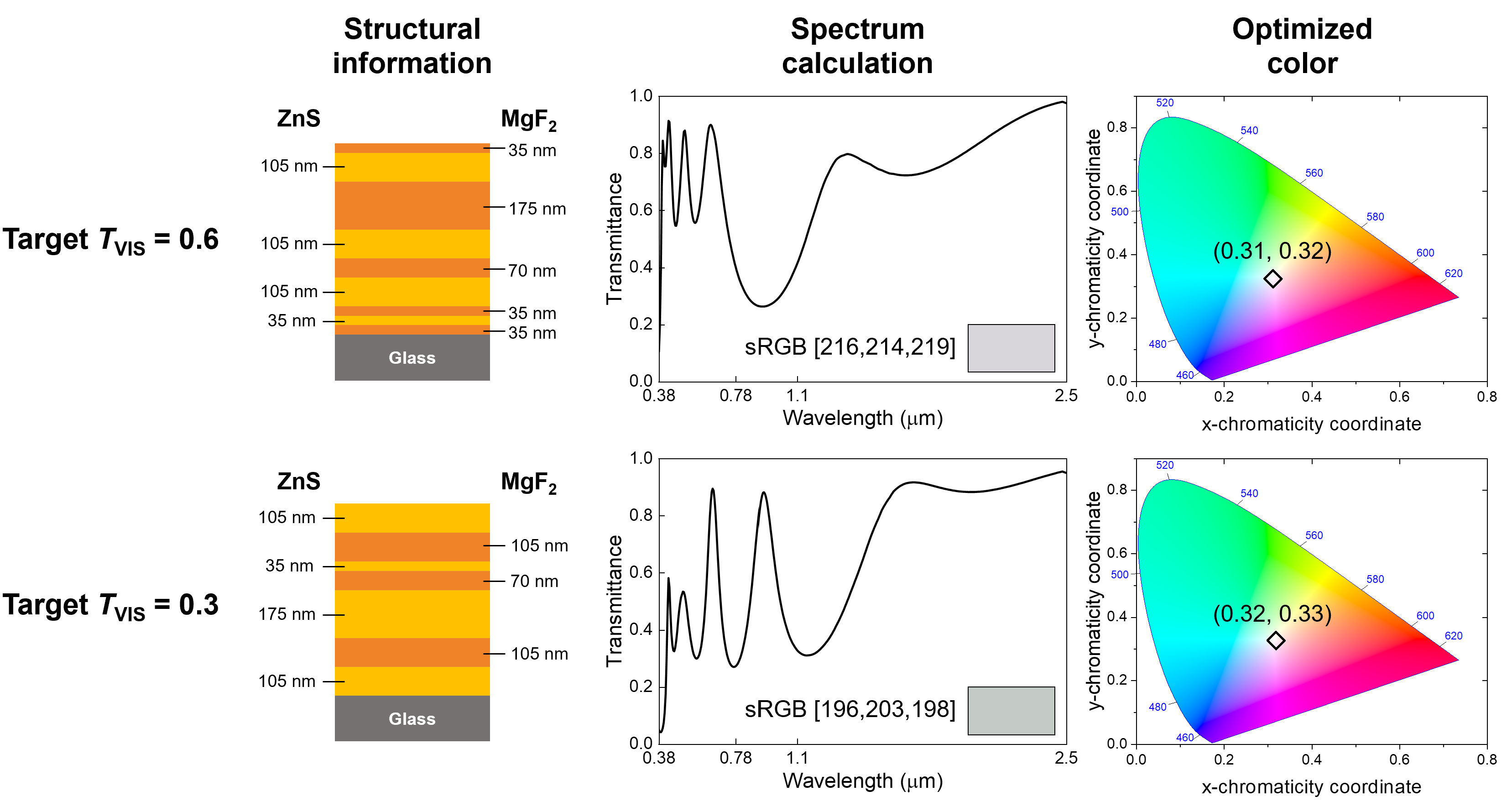


**Figure S6. Design of neutral-colored cooling windows.** Top: Target *T*_VIS_ = 0.6 with neutral color. Bottom: Target *T*_VIS_ = 0.3 with neutral color. Left: Structural information. Middle: Transmittance spectra of the multilayers. Right: Color coordinates corresponding to the transmittance spectra. Both designs aim for a color coordinate of (x, y) = (0.33, 0.33).
